# Reprogramming the inactive X chromosome: dynamics and insights from the germline

**DOI:** 10.1042/BST20250549

**Published:** 2026-03-24

**Authors:** Yolanda Moyano Rodriguez, Maud Borensztein

**Affiliations:** IGMM, Univ Montpellier, CNRS, Montpellier, France

**Keywords:** dosage compensation, epigenetic reprogramming, methylation, monoallelic expression, primordial germ cells, X-chromosome inactivation

## Abstract

Germline reprogramming is an essential process that resets the epigenome prior to gamete formation. Primordial germ cells (PGCs), the progenitors of oocytes and spermatozoa, undergo extensive epigenetic remodelling during development, including genome-wide DNA demethylation, histone modification remodelling, and large-scale reorganisation of 3D genome architecture. In female mammals, an additional layer of epigenetic regulation occurs during PGC reprogramming: the reactivation of the inactive X chromosome, namely, X-chromosome reactivation (XCR). Female PGC precursors carry an inactive X chromosome to ensure dosage compensation prior to reprogramming. While X-chromosome inactivation has been extensively studied for decades, XCR has only more recently emerged as a focus of investigation, and its functional importance for germline development and reproduction remains unclear. XCR takes place along PGC differentiation, from early emergence to meiosis, and involves loss of the long non-coding RNA *XIST/Xist* coating, DNA demethylation at X-linked promoters, and re-expression of X-linked genes from the inactivated X. Sequential molecular events occurring during XCR have been characterised using both *in vivo* and *in vitro* approaches in a broad range of mammals from rodents to humans. In recent years, the emergence of low-input and single-cell omics technologies has substantially advanced our understanding of the inactive X-chromosome reactivation in the germline. In this review, we synthetise recent insights into XCR dynamics in mouse, human, and non-human primate PGCs. We discuss the remaining knowledge gaps and the future perspectives in the field of XCR and germline epigenetic reprogramming.

The germline is unique among cell types, as it alone forms the seed for the next generation, transmitting genetic and epigenetic information. Accordingly, germ cells undergo profound epigenetic remodelling genome-wide, allowing the acquisition of a totipotent state in the future zygote, which is crucial for appropriate development. Female primordial germ cells (PGCs), the earliest precursors of the germline, undergo an additional step of reprogramming characterised by the loss of X-chromosome inactivation (XCI), hereafter called X-chromosome reactivation (XCR).

During female embryonic development, one of the two X chromosomes is transcriptionally inactivated in somatic lineages to compensate for X-linked gene dosage. This transcriptional inactivation, mediated by XCI, is lost in PGCs. However, the importance of XCR in PGC development and reprogramming, or later in gametogenesis, remains elusive.

Germline specification in mammals occurs during early gastrulation, when the PGC population appears [1,2,3,4,5]. PGC development encompasses migration, gonadal colonisation, proliferation, epigenetic reprogramming, and sex determination (Figure 1) [2,6]. The transcriptional network of the germline is established during specification, while the somatic programme is repressed, accompanied by extensive chromatin reprogramming. PGC reprogramming includes global DNA demethylation, erasure of genomic imprinting, remodelling of histone marks, and, in females, XCR. This epigenetic resetting allows the acquisition of the sex-specific epigenetic landscape in gametes [7–10].

**Figure 1:**
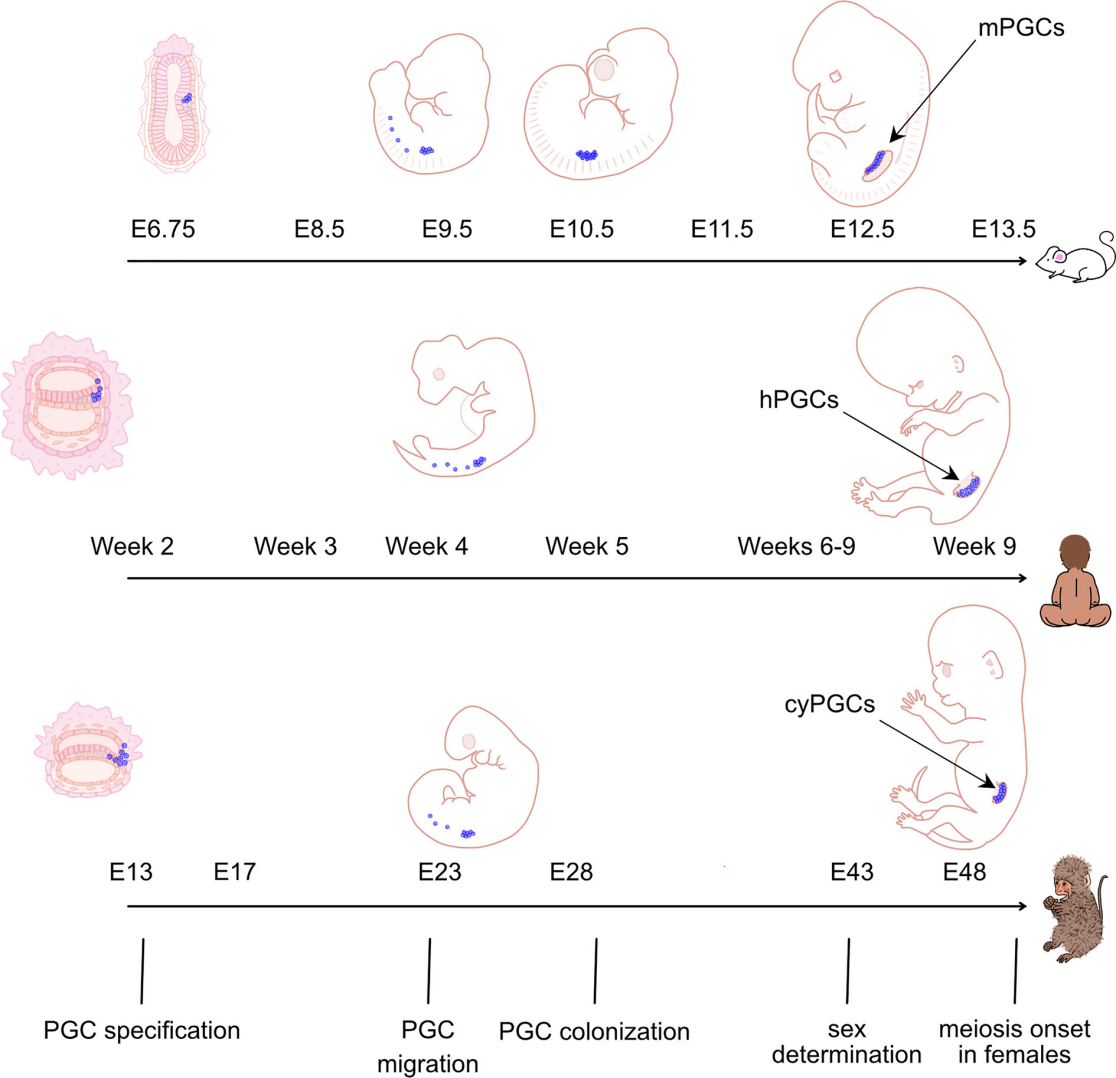
PGC specification during mouse, human, and macaque embryo development Schematic representation of mouse (top), human (middle), and macaque (bottom). PGCs (shown in blue) from specification to meiosis onset during embryo development. These events follow a broadly similar trajectory in eutherians despite differences in their developmental origin [11], [1,2,3,4,5]. Upon specification, a small PGC population migrates through the hindgut to colonise the genital ridge (future gonads), where the population expands prior to sex differentiation [2,6]. Subsequently, female PGCs switch from a mitotic to a meiotic cell cycle and arrest in prophase I until puberty [9,2].

Despite extensive knowledge of genome-wide reprogramming in the germline, the relevance of epigenomic and transcriptional changes at the X-chromosome level remains poorly understood. The PGC population is scarce at early stages and difficult to assess *in vivo*, especially in humans. However, the development of low-input and single-cell transcriptomic and genomic techniques, combined with *in vitro* germline systems, has allowed major advances in the study of germline reprogramming at an unprecedented pace, including recent insights into XCR dynamics in mice and macaques [12–16].

In this review, we discuss the reprogramming events occurring during XCR in the female germline in mammals, with a focus on recent insights in humans, monkeys, and mice (Figure 2). We also attempt to infer the relevance of this phenomenon in female germline reprogramming.

**Figure 2:**
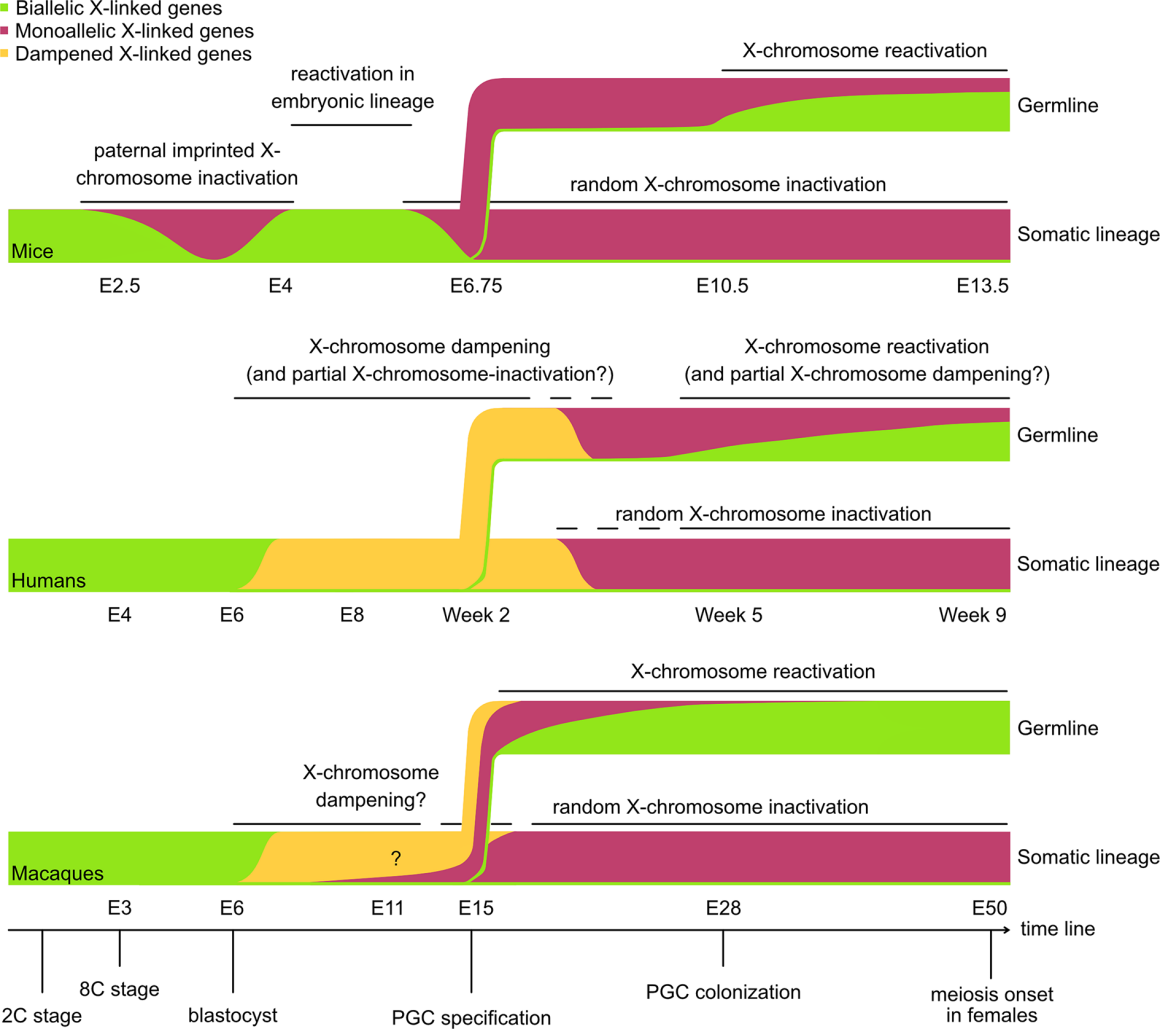
X-chromosome transcriptional dynamics during mammalian embryonic development X-linked gene allelic expression (biallelic in green and monoallelic in dark pink) is shown during embryonic development in mice (top), humans (middle), and macaques (bottom). Top: In rodents, two waves of XCI occur during development. First, paternal imprinted XCI (iXCI) initiates shortly after zygotic genome activation (ZGA), at the 4- to 8-cell stage, and progresses during blastocyst development [17,18,19,20]. iXCI is followed by XCR in the inner cell mass (ICM) from embryonic day (E) 3.5 to E4.5 [17,21], whereas the paternal Xi is maintained in extraembryonic tissues (not shown) [22,23,24]. A second event of XCI, random XCI (rXCI), affects either the maternal or paternal X chromosome in the epiblast around E5.5 [25]. In the germline, specified at E6.75, XCR starts upon loss of *Xist* expression around E9.5, and biallelic expression of X-linked genes is detected from E10.5. Middle: In human peri-implantation blastocysts (E4 to E7), X-linked transcription is reduced from both X chromosomes through a process known as X-chromosome dampening (XCD, in yellow) [26,27]. X-linked transcriptional activity progressively decreases, becoming predominantly monoallelic from week 4 of development [28]. In humans, PGCs are specified during XCI completion (week 2), and it is presumed that XCR occurs approximately two weeks later, concomitant with *XIST* loss. Bottom: In macaques, XCI initiates at peri-implantation stages (E9) and is completed by gastrulation (E15) [14,29], with no evidence for XCD based on current analyses. PGCs emerge between E11 and E17, also prior to full XCI, as in humans. In this case, XCI establishment and XCR overlap temporally, as XCR is proposed to initiate around E15. Question marks are included for X-chromosome dampening, as its existence remains under debate in the human germline and in the early development of non-human primates.

## To reactivate, we need to prior inactivate

XCI was first proposed 65 yr ago by Mary Lyon [30] and has been extensively studied since then [31–33,19,20,22,23,24]. XCI consists of the transcriptional silencing of an entire X chromosome, with some genes escaping silencing, known as ‘escapees’. XCI is accompanied by X-chromosome up-regulation (XCU), which promotes the transcriptional up-regulation of X-linked genes to balance expression with autosomes when a single active X chromosome (Xa) is expressed in a diploid cell (X:A ratio ∼1) [17, 34–38].

In mice, XCI is initiated by the long non-coding RNA (lncRNA) *Xist*, which coats in *cis* the future inactive X chromosome (Xi) [39–43] (Figure 2). *Xist* triggers transcriptional repression through the recruitment of the RNA-binding protein SPEN and the ribonucleoprotein HNRNPK, which scaffold chromatin-remodelling complexes, including the histone deacetylase HDAC3 [42–54]. Additionally, the Xi acquires a specific 3D conformation that differs from the Xa and autosomes. While the Xa is organised into topologically associating domains (TADs), the Xi is less structured, with reduced TADs, and becomes compacted into two large unorganised domains, called ‘megadomains’ [55–63]. During XCI, RNA polymerase II and components of the transcriptional machinery are rapidly excluded, promoting gene silencing before the establishment of a stable repressive landscape including Polycomb-mediated repressive marks H3K27me3 and H2AK119ub, and DNA methylation at promoters [31,63–66]. Once XCI is established, it is stably maintained and propagated through cell divisions throughout life in somatic cell lineages; however, this silencing is reversed in the female germline, where XCR occurs as part of germline epigenetic reprogramming.

Since its discovery in mice, XCI has been investigated in other mammals, revealing species-specific XCI characteristics. In humans, XCI seems to initiate around implantation, probably in extraembryonic tissues as seen in cynomolgus monkeys [14,44,29]. Prior to XCI, dosage compensation is achieved by reduced X-linked gene transcription, with *XIST* coating both X chromosomes, a process described as dampening [44,26, 27, 67–69]. Despite some discussion on the existence of dampening, both dampening and X-chromosome inactivation have been proposed as mechanisms of dosage compensation in early human development. Current data suggest that these mechanisms may reflect distinct developmental contexts [44,70,71]. (Figure 2). In addition, a hominid-specific lncRNA *XACT* coats the Xa in early human embryos, naïve pluripotent stem cells, and PGCs, but recent loss-of-function studies indicate that XACT is dispensable for XCI, leaving its functional role unresolved [44,27,69,72,73].

## Transcriptional awakening of the inactive X chromosome

Despite the importance of maintaining proper X dosage in the soma, XCR has been described in different developmental and experimental contexts. In the mouse ICM of the blastocyst, XCR occurs rapidly, within less than 24 h, and is initiated for a subset of genes despite the presence of an *Xist* cloud, whereas other genes reactivate later and only after the loss of *Xist* coating and H3K27me3 enrichment [74]. By contrast, during somatic cell reprogramming to induced pluripotent stem cells (iPSCs), XCR occurs progressively over several days, concomitant with *Xist* loss and gradual acquisition of biallelic expression of X-linked genes [75–79]. Finally, *in vitro* germline models such as PGC-like cells (PGCLCs) recapitulate key aspects of germline X-chromosome dynamics, although the XCI-XCR kinetics may differ, which should be considered when translating mechanistic requirements from *in vitro* to *in vivo* [12,14–16,29,80–82].

PGCLCs are generated *in vitro* by stepwise differentiation of embryonic stem cells or iPSCs through an epiblast-like cell intermediate, followed by germline specification using defined cytokine signalling. These cells transcriptionally and epigenetically resemble early migratory PGCs. Under extended culture conditions, PGCLCs can be further differentiated towards oogonia-like cells and undergo meiosis, providing an experimental system to study later stages of germline development (reviewed in [7,83–85]).

**Figure 3:**
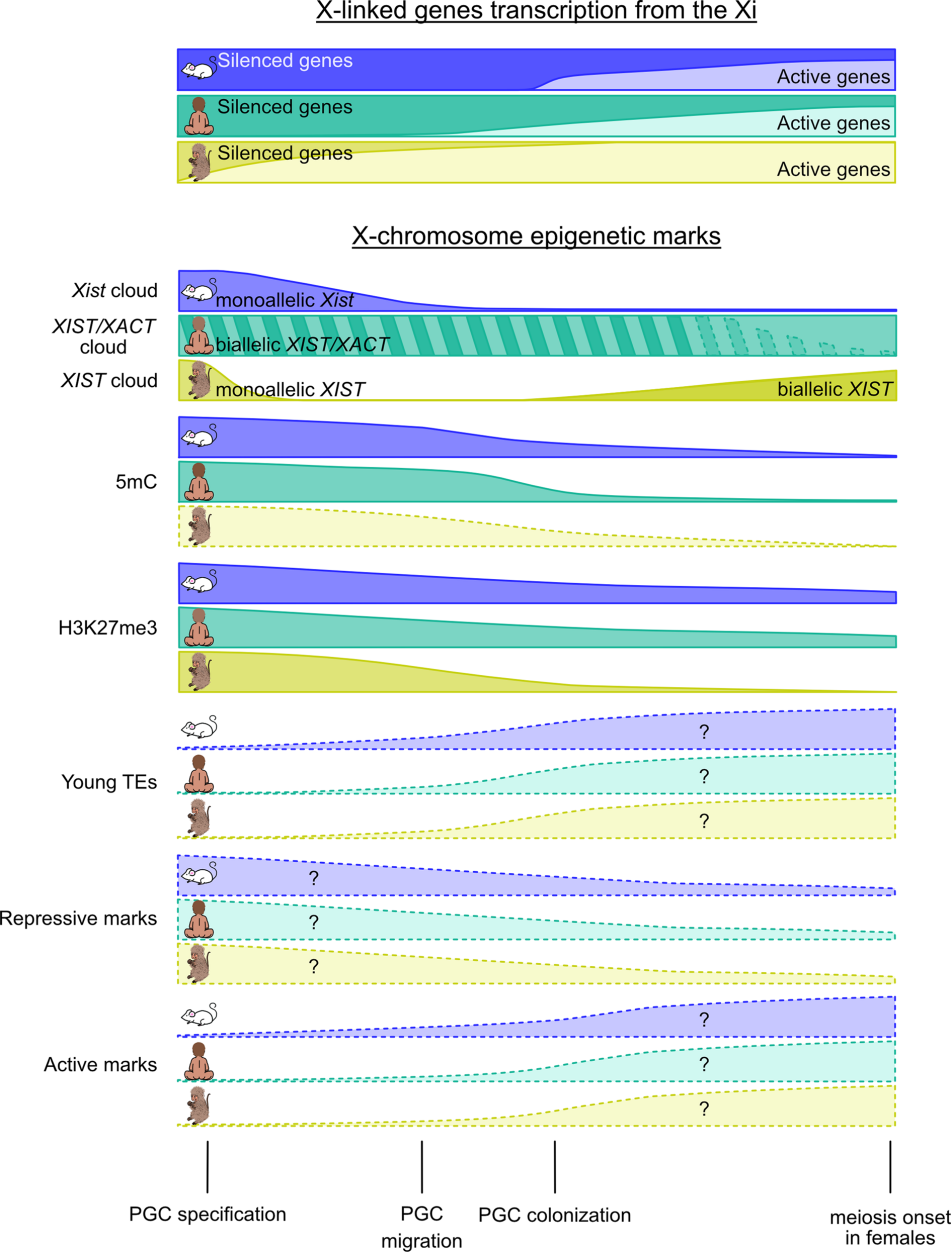
PGC specification and X-chromosome chromatin landscape Top: Panel represent mouse (top; purple), human (bottom; dark green) and macaque (bottom; light green) X-linked gene expression from the Xi during PGC development, based on [12–15,26,28,90]. Middle: Shown are changes in *Xist/XIST* expression (mouse, human and macaque [14,29,26,88,91,92,93]), *XACT* expression (human only [26]), DNA methylation (5mC), H3K27me3 enrichment [12,14,15,28,94,90,95,11], young transposable element (TE) expression, and global remodelling of repressive and active histone marks. DNA demethylation trends are supported by *in vivo* data in mouse [15] and human [94,96], whereas in macaque they are inferred from *in vitro* germline models and H3K27me3 changes [80]. Young TE activation in mouse and human is inferred from genome-wide expression analyses during germline reprogramming [28,97,98,99], while corresponding data are currently unavailable for macaque. Predicted changes in additional histone marks are based on published X-chromosome-specific or genome-wide chromatin profiling data, although most studies lack allele-specific resolution [94,100,101,96,95,102]. Question marks indicate unknown or unresolved dynamics, and lighter shading denotes trends inferred from *in vitro* or non-allele-specific evidence. Bottom: Schematic representation of major events occurring in PGC differentiation.

Defining XCR dynamics and timing in mouse PGCs (mPGCs) was long limited by the technical constraints, including the low number of allele-informative X-linked genes that could be assessed using earlier targeted expression assays, such as allele-specific PCR-based approaches, which rely on the presence of polymorphisms to distinguish maternal and paternal alleles [86–88]. As a consequence, initial studies reported incomplete reactivation timing of X-linked genes. Recently, chromosome-wide analyses have characterised XCR dynamics during mPGC development from E9.5 to E16.5 [12,15]; discussed in [89]. These studies, based on allele-specific single-cell RNA sequencing in mouse hybrid crosses, have determined that XCR starts from E9.5, with *Xist* being no longer expressed [12,15]. Gonadal mPGCs then progressively reacquire biallelic expression of X-linked genes from E10.5 onwards. Based on their reactivation dynamics, X-linked genes can be broadly classified as early- (biallelic from E10.5), intermediate- (biallelic from E11.5), late-reactivated (biallelic from E12.5), or very late reactivated genes, the latter remaining monoallelically expressed beyond meiosis onset and only reactivating between E13.5 and E16.5 [12,15] (Figures 2 and 3).

In contrast, XCR dynamics in the human germline remain less well defined, mostly due to limited access to tissues and to a small number of allele-informative genes, often compounded by the lack of polymorphisms and parental haplotype information. The first RNA-seq studies observed XCR in migratory human PGCs (hPGCs) at early developmental stages (week 4), consistent with previous RNA-FISH observations [26,28]. These findings were reinforced by the observed increased X-linked gene expression in female PGCs compared with males, the presence of unmethylated X-linked promoters, and the loss of the characteristic H3K27me3 focus associated with the Xi [26,94,91]. However, subsequent analysis using higher SNP coverage and parental haplotype information showed that XCR in humans occurs progressively and is not fully completed even long after meiosis onset (week 17) [90] (Figures 2 and 3). Another layer of complexity arises from the coexistence of XCU and XCR in early PGCs (X:A>1), observed both *in vivo* and *in vitro*, independently of sex, in mice and primates [13,38,92,103]. Different genes may have different contributions to XCU-chromosome up-regulation and therefore complicate the assessment of completed XCR if not assessed at the allelic level [38].

In monkeys, biallelic expression of a subset of X-linked genes was detected by nascent RNA-FISH in pre-migratory cynomolgus monkey PGCs (cyPGCs) and progressively increases during migration, becoming complete before meiosis entry (E50) [13,14] (Figures 2 and 3). Together, these observations highlight species-specific features of XCR, particularly regarding the timing and extent of complete XCR [12–14,26,90]. These findings open important questions: whether early-reactivated genes need to be expressed biallelically to support germline development and/or reprogramming, whether only a subset of X-linked genes need to be dosage compensated, and whether XCR may influence meiosis in a species-specific manner.

Despite interspecies differences, some X-linked genes show similar reactivation dynamics in different contexts, such as in the ICM of the blastocyst and in mPGCs in mice [15,74,21,18,25]. However, whether these shared dynamics rely on common regulatory requirements remains unknown. In the following sections, we will discuss the molecular events occurring during XCR in PGCs and explore potential principles across mammalian species and developmental contexts.

## Role of the lncRNA *Xist* in X-chromosome reactivation

Loss of functional *XIST/Xist* coating leads to variable degrees of XCR in a cell-type-dependent manner, suggesting an important role of *Xist* in repressing XCR, as well as the involvement of additional factors for complete XCR [53,104–106]. In the mouse ICM, early reactivated genes become biallelically expressed despite the persistence of an *Xist* RNA cloud and are characterised by a lack of H3K27me3 enrichment at their promoters, the presence of MYC and YY1 transcription factor binding sites, and enrichment of the active H3K4me3 marks [74,107]. In contrast, late-reactivated genes become biallelic only after the loss of *Xist* coating and active demethylation of H3K27me3 [74]. We now wonder what the role of *Xist* is in PGCs, considering that XCR takes days in PGCs compared with hours in ICM and only occurs after *Xist* is lost in mice.

In mice, the *Xist* cloud is present in early mPGCs and is progressively lost between E8.5 and E10.5 [88,91,92,93] (Figure 3). Loss of *Xist* coating represents the earliest detectable event of XCR in mPGCs, although whether it is strictly required for proper reactivation remains unknown [15,86,88,92] (Figure 3).

In monkeys, following specification, most cyPGCs show *XIST* coating on one X chromosome, which is progressively lost during migration. In contrast with other species, both female and male cyPGCs subsequently reacquire *XIST* expression, although it remains as a dispersed cloud, with no apparent impact on X-linked gene expression [14,29] (Figure 3).

In the case of humans, *XIST* and *XACT* are expressed and coat both X chromosomes from early to meiotic hPGCs, with *XIST* expression being progressively attenuated from week 7 and lost by meiosis [26] (Figure 3). Notably, XCR starts before complete *XIST* loss and, as in mice, coincides with increased X-linked gene expression compared with autosomes (X:A ratio >1) [26,71,97]. The role of *XIST* in humans is particularly intriguing, as both male and female X chromosomes are coated by *XIST* in pre-implantation embryos despite the X being dampened only in females [26,27]. In female hPGCs, *XIST* coating persists but is attenuated, and biallelic X-linked gene expression is dampened [26] (Figure 2). This dampening may be difficult to distinguish from incomplete XCR when assessed at the chromosome-wide rather than single-gene-level analyses, leaving the contribution of *XIST* to XCI, dampening, or reactivation in hPGCs unresolved.

Together, these observations suggest that *Xist/XIST* may have species-specific modes of action during XCR. In pluripotent cells, *Xist* expression is tightly controlled by pluripotency TFs and lncRNAs, such as NANOG, PRDM14, and *Ftx* [108–112]. In addition, interferon γ pathway activation during iPSC reprogramming promotes *Xist* down-regulation and XCR [75]. Whether similar regulatory mechanisms operate in the germline, particularly in humans, remains largely unknown. Naïve pluripotency factors expressed in PGCs, together with the down-regulation of primed pluripotency-associated factors (such as OTX2), may be involved in *XIST/Xist* down-regulation, but this remains to be elucidated in the germline.

## DNA demethylation, PGC reprogramming, and X-reactivation

One of the major mechanisms contributing to the maintenance of XCI is DNA methylation (DNAme). During female germline development, XCR occurs in the context of profound DNAme erasure. Accordingly, DNAme loss and XCR occur progressively and simultaneously in PGCs. PGCs are the cell type in the body with the lowest methylation levels, with approximatively 6% of remaining methylated CpGs around meiotic onset [28,94,97,100, 113, 114].

Throughout PGC development and migration, the genome is demethylated by combined active and passive mechanisms, with only a few exceptions—gene bodies, young and active transposable elements, satellite repeats at centromeric and pericentromeric regions, and a subset of promoters required for neuronal lineages [28,94,97,100, 101, 115–117]. Passive demethylation is particularly important for global demethylation in migrating PGCs [28,115,101,118,119]. It results from reduced activity of the DNA methyltransferases DNMT3A, DNMT3B, and DNMT1, combined with extensive cell proliferation, leading to dilution of the marks [28,94,97,117,120,98]. Upon arrival at the genital ridges, further DNA demethylation depends on active mechanisms involving TET demethylases and the base excision repair pathway [28,94,101,120,121]. However, these pathways do not fully account for the extent of DNA demethylation in PGCs, potentially accompanied by additional mechanisms. Importantly, how these global DNA demethylation impacts XCR in PGCs remains poorly understood.

Evidence from female iPSC reprogramming indicates that the lack of DNMTs combined with an *Xist*-inducible deletion induces premature XCR [76,77]. Although global DNA methylation levels in the epiblast are lower than in differentiated somatic cells, these results suggest that DNA methylation could contribute to the maintenance of X-linked gene silencing prior to reactivation and that its loss may be required for efficient XCR during PGC differentiation.

In mice, CpG island demethylation occurs progressively from E9.5 to E13.5, also concomitant with XCR [115,116,122] (Figure 3). Despite undergoing extensive demethylation, the X chromosomes remain more highly methylated than autosomes by E13.5. Regions displaying the highest methylation levels at earlier developmental stages, or enriched for LINE-1 elements, are resistant to demethylation in late mPGCs [28,97,116,98,99]. When focusing especially on X-linked gene promoters, we showed that all X-linked reactivation classes (early, intermediate, late, and very late) display low DNA methylation levels in E10.5 mPGCs compared with the E6.5 epiblast, although escapees and early-reactivated genes show the lowest levels prior to the onset of demethylation [15,101]. Moreover, we were able to demonstrate that the low persistent methylation levels were exclusively in the Xi [15]. Together, this suggests the idea that DNAme loss may be required for efficient X-linked gene reactivation.

For primates, allele-specific information on DNAme at the X chromosomes comes from *in vitro* cyPGCLCs and hPGCLCs [76,78]. These studies observe that the Xi displays lower methylation levels than observed in *in vivo* mouse PGCs while still retaining higher methylation levels than the Xa. Data generated without distinction between the Xi and Xa can nevertheless provide complementary information into DNAme dynamics alongside current knowledge of XCR dynamics. In human PGCs, CpG island promoters become low methylated by week 5.5—during XCR and further demethylated by weeks 9 and 11 [94,96] (Figure 3). Lower methylation levels observed in humans could be attributed, at least in part, to lower density of LINE-1, although species-specific methylation landscapes or additional regulatory factors cannot be excluded [123–125].

Overall, despite clear differences in DNA demethylation dynamics among mammals, the specific contribution of DNA demethylation to XCR remains difficult to assess due to the absence of sex- and allele-specific information at the Xi and Xa chromosome scales in PGCs.

## H3K27me3 and the resistance to XCR

At the genome-wide level, H3K27me3 is associated with CpG-rich bivalent or ‘poised’ promoters of developmental genes, where it mediates CpG island-related gene silencing. It also contributes to the regulation of differentiation programmes and transposable elements (TEs), with a sex-specific distribution emerging in PGCs [122,126–130]. However, H3K27me3 distribution differs considerably between the X chromosome and autosomes [31,131]. During PGC development, early PGCs progressively accumulate H3K27me3 genome-wide while migrating towards the genital ridges [86,88,94,93,95, 132, 133] (Figure 3). Although this histone mark is continuously deposited, its global level is transiently reduced between E10.5 and E12.5 in mice through the activity of X-linked H3K27 demethylase KDM6A/UTX. Similar reductions in H3K27me3 levels have also been reported in other mammals, such as in humans, where global H3K27me3 decreases in hPGCs before sexual differentiation [28,94,90,100,95]. In pigs, similar trends could be expected with *Kdm6a* expression being higher in migratory PGCs than in somatic cells [126,134,11]. Later, H3K27me3 is newly re-acquired in meiotic germ cells [113,100,126,135] (Figure 3).

H3K27me3 is deposited by the Polycomb repressive complex 2 (PRC2), whose subunits are detected in PGC nuclei of both sexes, at least in mice [93,126,127,132,135,136]. However, loss of PRC2 activity at E13.5 has a greater impact in female than in male PGCs, leading to derepression of meiotic genes and transposable elements, and is accompanied by increased DNA damage specifically in females [126,127]. This may reflect the fact that in males, TE repression upon DNA demethylation is regulated primarily by another repressive histone mark, H3K9me3, although PRC2-dependent regulation may also become relevant later during meiosis [126,130]. Interestingly, during the transition from E13.5 male mPGCLCs to pro-spermatogonia, the promoters of coding genes are more enriched for H3K27me3 on the X than on autosomes, and TEs and the X-linked genes become deregulated upon loss of this mark [127].

In mice, H3K27me3 foci associated with the Xi are detected by immunofluorescence in nascent PGCs and are lost from E9.5 onwards, concomitant with the disappearance of the *Xist* cloud [86,88,92,93,132,136] (Figure 3). In contrast, H3K27me3 dynamics is more variable in primates. In humans, early hPGCs start losing H3K27me3 foci from week 5, despite *XIST* still being present, and the mark is largely absent before meiosis entry [14,28,94,90,95,11]. In cynomolgus monkeys, H3K27me3 foci persist up to E23-28 despite XIST being lost prior to PGC migration and are then completely absent in meiotic cells [14] (Figure 3).

H3K27me3 distribution in the X chromosome has been studied in mice using ultra-low-input chromatin profiling approaches in mPGCs from E11.5 to E16.5 [12,15]. These studies revealed that H3K27me3 is enriched at promoters of inactive X-linked genes that are not yet reactivated and is progressively lost as genes undergo reactivation [12,15] (Figure 3). Notably, early-reactivated genes and escapees show enrichment of H3K27me3 within gene bodies, while late-reactivated or non-reactivated genes retain the mark at their promoters [15]. In addition, reanalysis of published datasets shows that loss of the PRC2 catalytic subunit EZH2 may lead to differential expression of X-linked genes, although allele-specific effects could not be resolved [12,126].

Together, these data highlight a strong correlation between H3K27me3 and XCR in PGCs. However, direct mechanistic evidence demonstrating whether H3K27me3 removal is required for XCR in the germline, as shown in the ICM of the blastocyst, is still lacking [31,74]. Moreover, comparable allele-specific analyses distinguishing between the active and inactive X chromosomes are still largely missing in primates and humans.

## Histone remodelling beyond H3K27me3

The PGC genome undergoes a series of histone modifications during specification in addition to H3K27me3 remodelling. This chromatin reshuffling includes the gain of active histone marks (H3K4me3 and H3K27ac), the loss of repressive histone marks (H3K9me2/3 and H2K119ub), and the remodelling of bivalent marks [8,100,122,126].

In humans and mice, heterochromatin-associated marks—H3K9me2, H3K9me3, MacroH2A2, and HP1α—are globally reduced in PGCs compared with somatic cells. During specification, these marks progressively decrease from migrating to sexually differentiated PGCs [28,97,113,98,95]. In contrast, *de novo* acquisition of H3K9me3 occurs at a subset of demethylated promoters of germline genes, in bivalency with the active mark H3K4me3, thus pausing their reactivation [100,98,96,129,137]. Promoters for genes escaping DNA demethylation are often co-occupied by 5mC and H3K9me3. Examples include silenced young TEs, such as LINE-1, SVA, and ERVK, as well as satellite repeats, which are also enriched for MacroH2A2 and HP1α [28,97,96,126,130,138]. Finally, the Polycomb-associated mark H2AK119ub has also been extensively studied during human and mouse PGC reprogramming [100,101,102]. This histone mark is globally reduced by week 9 in hPGCs, especially on the X chromosome in females, which instead retains relatively higher 5mC levels at X-linked CpG island promoters compared with autosomes [100]. However, these studies were not performed in an allele-specific manner (Xi versus Xa), in contrast with H3K27me3 analyses, making it difficult to determine the precise contribution of these different histone marks’ remodelling to XCR in PGCs.

By contrast, active marks, H3K4me1, H3K4me3, H3K9ac, and H3K27ac, are globally enriched in PGCs compared with somatic cells [28,100,96,95]. This enrichment occurs at promoters and enhancers of up-regulated germline genes, including *DAZL*, *PRDM1*, and *PRDM14*, as well as derepressed TEs [118,133]. Among TEs, LINE-1 becomes transcriptionally activated from E10.5 in mPGCs, coinciding with their enrichment in H3K4me3 [28,97,98,99] (Figure 3).

TE-associated chromatin signature has been proposed to influence XCR, as previously suggested for XCI, raising the possibility that TE density or activity could impact X-linked gene reactivation dynamics [123–125,139,140]. However, no specific enrichment of LINE-1 was observed in *Xist* entry sites, which are the first regions bound by *Xist*, when compared with the entire X [15]. Interestingly, recent work suggests that TEs within the X chromosome may undergo specific dosage compensation, raising the possibility that TE activity could contribute to XCI and XCR [141].

Despite significant progress in defining the global distribution of histone marks during PGC development, the allele-specific dynamics of most histone marks on the active and inactive X chromosomes remain largely missing, with the notable exception of H3K27me3 in gonadal female PGCs.

## Chromosomal architecture role in gene expression

The 3D architecture of the X chromosome may contribute to XCR. Genomic localisation has been suggested to influence reactivation dynamics, with genes reactivating earlier when located near escapees and later when closer to the *Xist* locus [15,76,79]. Escapees show a more organised 3D structure within the two megadomains, with preserved TADs, compared with inactivated genes and are localised in the vicinity of the Xi [59,65]. Furthermore, the TADs lost during XCI are reacquired upon reprogramming in mouse iPSCs [79,142]. Additionally, Xi has been observed to be more compacted than Xa in somatic cells [143–146].

In PGCs, a recent preprint reports reduced intra-chromosomal contacts in gonadal PGCs compared with soma, consistent with a decompaction of the X chromosome upon XCR [147]. However, whether such 3D differences between PGCs and soma are acquired before, concomitantly, or as a consequence of reactivation remains unknown, as information on earlier stages is missing. Supporting a link between chromosome organisation and XCR, the Xa decompaction and reactivation of an X-linked reporter in mouse iPSCs was compromised by the lack of the cohesin subunit SMC1A [144]. Moreover, lack of histone acetyltransferase complexes was shown to reduce Xa decompaction in somatic cells [145]. However, the causal contribution of X-chromosome conformation to XCI establishment, maintenance, or reactivation remains debated [59,61,62,65,144].

At a smaller scale, chromatin accessibility increases upon XCR during iPSC reprogramming in both *in vivo* PGCs and *in vitro* PGCLCs [78,79,103,148–150]. Increased accessibility may facilitate transcription factor binding and contribute to X-linked gene reactivation [74,76,78,112,136,142,148]. Recent work further identified female-specific accessible regions on both autosomes and the X chromosome in human germ cells, suggesting the presence of potential cis-regulatory elements relevant for sex differentiation and/or XCR in the germline [103].

## Final remarks

Despite significant progress made in recent years, our understanding of XCR in the germline remains incomplete, especially regarding its regulation and functional relevance. A major outstanding question concerns the role of XCR during germline reprogramming and its impact on fertility. Defects in gamete epigenetic reprogramming compromise fertility and embryonic development, both *in vivo* and *in vitro* [151–154].

Insights from sex chromosome aneuploidies further highlight the importance of balanced X-linked gene dosage in the germline. Turner syndrome patients (XO, 45) display infertility and additional somatic defects in humans, whereas XO female mice are predominantly subfertile [155–157]. Recently, analysis of human germ cells from Klinefelter syndrome (XXY, 47) embryos showed that the presence of an extra X chromosome leads to an increased X/A expression ratio compared with XY males and XX females. This altered X-chromosome dosage is associated with delayed germline differentiation, a reduced pool of mature germ cells, aberrant *XIST*/*XACT* coating, lower DNA methylation levels, and reduced PGC migration compared with XX females and XY males [158]. Together, these findings strongly suggest that precise regulation of X-linked gene expression is critical for germline development. Consistent with this idea, *in vitro* studies show that PGCLCs failing to undergo XCI during differentiation are unable to activate the meiotic programme upon oocyte induction [16]. In contrast, PGCLCs that undergo XCI followed by XCR acquire an advanced meiotic state, suggesting that X-chromosome dosage is important for germline progression [16]. Altogether, these studies further underscore the importance of understanding the relevance of XCR and X-linked gene dosage in the germline.

## Perspectives

- Determining how X-chromosome transcriptional activity influences germline identity, sexual dimorphism, and reproductive capacity will be essential for improving fertility treatments in the future.
- Recent works have delineated the sequential molecular events accompanying XCR; however, whether these events are causative or permissive remains unknown.
- Future progress will rely on identifying the key regulators of XCR and assessing their impact on both XCR and germline fate. Achieving this will require the integration of low-input genomic approaches *in vivo* with advanced *in vitro* germline systems, particularly to overcome the current limitations in human studies [85,159].

## Open Access

Open access for this article is provided via our Subscribe to Open programme, made possible through the support of our subscribing institutions. Learn more here: https://portlandpress.com/pages/open_access_options_and_prices#conditional.

## Abbreviations

cyPGCs: cynomolgus monkey primordial germ cells
hPGCs: human primordial germ cells
ICM: inner cell mass
iPSCs: induced pluripotent stem cells
iXCI: imprinted X-chromosome inactivation
lncRNA: long non-coding RNA
mPGCs: mouse primordial germ cells
PGCLCs: primordial germ cell-like cells
PGCs: primordial germ cells
rXCI: random X-chromosome inactivation
TADs: topologically associating domains
TE: transposable elements
Xa: active X chromosome
XCI: X-chromosome inactivation
XCR: X-chromosome reactivation
XCU: X-chromosome up-regulation
Xi: inactive X chromosome
ZGA: zygotic genome activation
